# Differential effects of teriparatide, denosumab and zoledronate on hip structural and mechanical parameters in osteoporosis; a real-life study

**DOI:** 10.1007/s40618-023-02280-4

**Published:** 2024-01-09

**Authors:** N. Jaarah, C. F. J. Lam, N. Lodhia, D. Dulnoan, A. E. Moore, G. Hampson

**Affiliations:** 1 Department of Chemical Pathology and Metabolic Medicine, St Thomas’ Hospital, 5Th Floor, North Wing, Lambeth Palace Road, London, UK; 2 Osteoporosis Unit, Guy’s Hospital, London, SE1 7EH UK

**Keywords:** Osteoporosis, Teriparatide, Denosumab, Zoledronate, Hip geometry

## Abstract

**Purpose:**

The aim of this study was to evaluate changes in hip geometry parameters following treatment with teriparatide (TPD), denosumab (Dmab) and zoledronate (ZOL) in real-life setting.

**Methods:**

We studied 249 patients with osteoporosis (OP) with mean [SD] age of 71.5 [11.1] years divided into 3 treatment groups; Group A received TPD; *n* = 55, Group B (Dmab); *n* = 116 and Group C (ZOL); *n* = 78 attending a routine metabolic bone clinic. Bone mineral density (BMD) was measured by DXA at the lumbar spine (LS), total hip (TH) and femoral neck (FN) prior to treatment and after 2 years (Group A), after a mean treatment duration of 3.3 [1.3] years (Group B) and after 1, 2 and 3 doses of ZOL (Group C) to assess treatment response. Hip structural analysis (HSA) was carried out retrospectively from DXA-acquired femur images at the narrow neck (NN), the intertrochanter (IT) and femoral shaft (FS).

**Results:**

Changes in** p**arameters of hip geometry and mechanical strength were seen in the following treatment. Percentage change in cross-sectional area (CSA): 3.56[1.6] % *p* = 0.01 and cross-sectional moment of inertia (CSMI): 4.1[1.8] % *p* = 0.029 increased at the NN only in Group A. Improvement in HSA parameters at the IT were seen in group B: CSA: 3.3[0.67]% *p* < 0.001, cortical thickness (Co Th): 2.8[0.78]% *p* = 0.001, CSMI: 5.9[1.3]% *p* < 0.001, section modulus (Z):6.2[1.1]% *p* < 0.001 and buckling ratio (BR): − 3.0[0.86]% *p* = 0.001 with small changes at the FS: CSA: 1.2[0.4]% *p* = 0.005, Z:1.6 [0.76]%, *p* = 0.04*.* Changes at the IT were also seen in Group C (after 2 doses): CSA: 2.5[0.77]% *p* = 0.017, Co Th: 2.4[0.84]% *p* = 0.012, CSMI: 3.9[1.3]% *p* = *0.017*, Z:5.2[1.16]% *p* < 0.001 and BR: − 3.1[0.88]% *p* = 0.001 and at the NN (following 3 doses): outer diameter (OD): 4.0[1.4]% *p* = 0.0005, endocortical diameter(ED): 4.3[1.67% *p* = 0.009, CSA:5.2[1.8]% *p* = 0.003, CSMI: 9.3[3.8]% *p* = 0.019*.*

**Conclusions:**

Analysis of the effect of OP therapies on hip geometry is useful in understanding the mechanisms of their anti-fracture effect and may provide additional information on their efficacy.

## Introduction

Osteoporosis is characterized by low bone mass, deterioration in skeletal micro-architecture and reduced bone strength which lead to an increased risk of fragility fracture. Its prevalence is increasing worldwide and it is estimated that 1 in 2 women and 1 in 5 men over the age of 50 years will sustain an osteoporotic fracture in their lifetime [1].

Osteoporosis medications include anti-resorptive and anabolic agents. Bisphosphonates (BPs) are the most commonly prescribed anti-resorptive osteoporosis drug and include oral preparations (alendronate, risedronate) and parenteral preparations (zoledronate) [2]. Denosumab (Dmab)) is a human monoclonal antibody that binds to receptor activator of nuclear factor kappa-B ligand (RANKL and is a potent anti-resorptive agent [2]. Trials of intravenous (iv) bisphosphonates; zoledronate (ZOL) have shown good anti-fracture efficacy with reduction in morphometric vertebral fractures, hip and non-vertebral fractures of 70%, 40% and 25%, respectively, following 3 years treatment with once yearly iv zoledronate [3]. Data from the FREEDOM trial, the pivotal trial of Dmab also showed significant reductions in fractures following 3 years of denosumab with relative risk reduction of 68% for vertebral fractures, 40% for hip fractures and 20% for non-vertebral fractures [4]. Teriparatide (TPD), the first approved anabolic agent, stimulates new bone formation. In the TPD phase 3 fracture prevention trial, treatment for a median time of 19 months reduced the risk of one or more vertebral fractures by 65% and 2 or more vertebral fractures by 77% and non-vertebral fractures by 35% [5]. The number of patients with a hip fracture was small and the trial was insufficiently powered to show any significant difference between TPD and placebo arms. Subsequent more recent meta-analysis showed that TPD reduced hip fractures by 56% in patients with osteoporosis [6].

Although both BPs and Dmab are anti-resorptive agents, their mode of action on the skeleton differ. In contrast to BPs, Dmab does not bind to mineral surfaces and can be more widely distributed through the skeleton and penetrate more deeply into cortical bone [7]. Changes in dual-energy X-ray absorptiometry (DXA)-derived bone mineral density (BMD) tends to plateau with BPs over time whereas with Dmab, increases in BMD is seen progressively as long as treatment is continued and is larger compared to BPs, although the effect on fracture risk reduction is not clear [7]. These anti-resorptive agents increase endocortical bone by reducing endosteal resorption pits and boosting mineralisation, thus reducing cortical porosity and increasing cortical thickness. Dmab was shown to increase hip and spine strength which occurred in both the trabecular and “cortical” compartments using finite element analysis (FEA) of hip and spine quantitative computed tomography (QCT) scans to assess hip and spine geometric parameters and strength, thus providing further insight into the anti-fracture risk effect of Dmab [8]. Other studies using high-resolution peripheral computed tomography (HR-pQCT) have shown that Dmab reduces cortical porosity more than alendronate and has a more potent inhibitory effect on bone remodelling, particularly in sites of cortical bone [9]. TPD affects the cortical and trabecular compartments differently. Large increases in BMD are seen in the axial skeleton at trabecular bone rich sites. Changes in BMD at sites rich in cortical bone are less. Although quantitative CT data show that TPD leads to a decrease in cortical volumetric BMD, it does not reduce structural parameters associated with bending strength at the femoral neck [10, 11].

Although measures of volumetric BMD in the cortical and trabecular compartments by HR-pQCT combined with the finite element analysis technique can provide useful insight into how the osteoporosis drugs above affect the biomechanical strength of the skeleton, it is not widely available in the routine clinical setting as it is used mainly in the research setting. HSA parameters which give a measure of hip geometry and strength can be derived from DXA scan images [12]. Hip structural analysis (HSA) using DXA-derived femur images within the analysis software has been shown to be useful in evaluating hip geometry and mechanical strength [12]. Although, HR-pQCT is considered the gold standard non-invasive technique for the assessment of bone quality, large epidemiological studies have shown that some hip geometric properties derived from DXA images can predict incident hip fractures [13, 14]. Studies using HSA have shown that Dmab and oral bisphosphonates (alendronate and risedronate) improved structural and mechanical parameters at the 3 femoral sites which included the narrow neck (NN), inter-trochanter (IT) and the femoral shaft (FS) [15, 16]. There is, however, a paucity of clinical data assessing the effects of the osteoporosis drugs on HSA parameters in a real-life setting and whether these additional parameters could prove useful in the assessment of treatment response. The aim of the study was to evaluate changes in hip structural and mechanical parameters following treatment with TPD, Dmab and ZOL in the setting of a metabolic bone clinic. This study proposed to assess whether these parameters may be used in the routine clinical setting to provide additional information on treatment response with the use of these commonly prescribed osteoporosis medications.

## Material and methods

### Subjects

We carried out a retrospective survey as part of service review, approved by the institution, of 249 patients with OP with mean [SD] age of 71.5 [11.1] years attending the metabolic bone clinic for their routine follow-up and who were or had been on parenteral osteoporosis medications. They were divided into 3 groups based on the drug therapy; Group A had received TPD; *n* = 55, F: 55, age: 73[8.3] years, Group B were on Dmab; *n* = 116, F: 105, M: 11, age 72.7[10.8] years and Group C were on intravenous ZOL; *n* = 78, F: 67, M: 11, age 68.5[12.8] years. Clinical information, bone mineral density (BMD) and biochemical measurements were obtained at their clinic visits. All patients on active osteoporosis drugs were advised to take vitamin D (800 IU daily) and calcium supplements (if their dietary calcium was < 700 mg/daily, assessed in clinic by dietary history or the use of an online calcium calculator).

The hospital’s electronic patient record system and medical records were used for data collection. This was carried out under close supervision by the senior clinical team. All data were anonymised and complied with the UK data protection act. BMD data at the lumbar spine (LS), femoral neck (FN) and total hip (TH) were obtained prior to starting treatment with teriparatide, denosumab or iv zoledronate (baseline values) and compared with values after 2 years of teriparatide (Group A), mean duration of treatment of (mean [SD]) of 39.6 [15.6] months with denosumab (Group B) and at 14.3 [4.0], 12.8 [1.8] and 13 [2.1] months following the 1st, 2nd and 3rd dose of zoledronate, respectively (Group C). Hip structural analysis (HSA) was carried out retrospectively from DXA-acquired femur images at the narrow neck (NN), the intertrochanter (IT) and femoral shaft (FS) at these time points. Percentage changes were derived. Routine laboratory measurements including estimated glomerular filtration rate (eGFR), albumin adjusted calcium, PTH concentration, 25(OH)vitamin D were done at baseline. Patients demographics, baseline BMD and biochemical data in the 3 groups are summarised in Table 1. Group C tended to be younger. Group A had significantly lower BMD at all 3 sites compared to Groups B and C. The prevalence of fractures was higher in Group A and B compared to Group C. There was no significant difference in the prevalence of secondary risk factors between the 3 groups. eGFR was lower and serum PTH higher in Group B.

**Table 1:** Summary of the demographics and baseline biochemical parameters of the study population

Parameters	Group A	Group B	Group C	*p* value
Mean (SD)	(Teriparatide) N = 55 F/M 55/0	(Denosumab) N = 116 F/M 105/11	(Zoledronate) N-77 F/M 66/11	
Age (years)	73.0 (8.3)	72.2 (10.8)	68.5 (12.8)	A vs B: *ns* A vs C: *ns* B vs C: *0.03*
Duration of follow up (month). Median (IQR)	24 (24, 30)	42 (30, 48)	40 (36, 47)	A vs B: < *0.001* A vs C: < *0.001* B vs C: *ns*
Number (%) with secondary causes of osteoporosis	17 (31)	44 (38)	29 (38)	*ns*
BMD lumbar spine (g/cm2)	0.67 (0.105)	0.79 (0.134)	0.777 (0.11)	A vs B: < *0.001* A vs C: < *0.001* B vs C: *ns*
T score lumbar spine	− 3.4 (0.97)	− 2.37 (1.22)	− 2.54 (1.01)	A vs B: < *0.001* A vs C: < *0.001* B vs C: *ns*
BMD total hip (g/cm2)	0.61 (0.109)	0.693 (0.099)	0.701 (0.123)	A vs B: < *0.001* A vs C: < *0.001* B vs C: *ns*
T score total hip	− 2.7 (0.9)	− 2.07 (0.78)	− 2.06 (0.93)	A vs B: < *0.001* A vs C: < *0.001* B vs C: *ns*
BMD femoral neck (g/cm2)	0.52 (0.081)	0.586 (0.093)	0.595 (0.089)	A vs B: < *0.001* A vs C: < *0.001* B vs C: *ns*
T score femoral neck	− 2.9 (0.736)	− 2.4 (0.82)	− 2.4 (0.81)	A vs B: < *0.001* A vs C: *0.024* B vs C: *ns*
Previous fractures n (%)	55 (100%)	96 (83%)	46 (59%)	A vs B: *ns* A vs C: < *0.001* B vs C: < *0.001*
Biochemical Parameters				
eGFR (mmol/L)	81.7 (22.5)	64.3 (26.3)	84.4 (22.6)	A vs B: < *0.001* A vs C: *ns* B vs C: < *0.001*
Adjusted calcium (mmol/L)	2.42 (0.12)	2.38 (0.1)	2.35 (0.09)	A vs B: *0.023* A vs C: < *0.001* B vs C: *ns*
PTH (ng/L)	40.2 (19.4)	52.5 (33.9)	38.7 (26.6)	A vs B: *0.022* A vs C: *ns* B vs C: *0.033*
25(OH)vitamin D (nmol/L)	68.0 (19.9)	74.2 (27.9)	62.7 (27)	A vs B: *ns* A vs C: *ns* B vs C: *ns*

*ns*  not significant. *p*  *< 0.05* is considered significant

### Dual energy X-ray absorptiometry (DXA) and hip structure analysis (HSA)

BMD at the lumbar spine (LS), total hip (TH) and femoral neck (FN) were measured by DXA on the Hologic Discovery scanner (Hologic, Inc. Bedford, MA). The CV for BMD measurement at the spine and total hip was 1.0% and 1.2%, respectively. The least significant change (LSC) in g/cm^2^ (%) of the LS spine is 0.022 g/cm^2^ (2.7%) and TH is 0.027 g/cm2 (3.4%). Quality-control scans were performed daily, using the anthropomorphometric phantom before the first patient was scanned. Before reporting any given BMD, the images were carefully assessed for patient positioning and DXA images artifacts by our experienced DXA technologists to avoid errors in acquisition and reporting. The DXA scan reports with the BMD results were issued for use in the clinical setting, only when all sources of possible errors were excluded.

Hip geometry analysis was performed using the HSA program using the femur image within the DXA analysis software. The regions of interest were recorded by a certified DXA technologists who carried the HSA analyses on all participants according to the standardised HSA analysis protocol. Accurate femur positioning had already been determined when the images for routine BMD measurements were acquired as detailed above. The program derives these following measurements at the NN, IT and FS: (1) subperiosteal (outer) width or diameter (OD) (2) endocortical (inner) width or diameter (ED) (3) cross-sectional area (CSA) which provides an index of resistance to axial forces, (4) estimated cortical thickness (Co Th), (5) cross-sectional moment of inertia (CSMI) which gives an estimate of resistance to bending forces and structural rigidity, (6) section modulus (Z) which is an indicator of bending strength, (7) buckling ratio (BR) which provides an estimate of susceptibility to local cortical buckling under compressive loads, (8) neck shaft angle (NSA), and (9) hip axis length (HAL) (12,172).

### Laboratory measurements

Routine laboratory biochemical tests including parathyroid hormone (PTH) were measured by standard laboratory methods on the Roche automated analysers (Roche diagnostics Limited, West Sussex, UK). eGFR was calculated from serum creatinine using the Modification of Diet in Renal disease formula [17]. Serum 25-hydroxy vitamin D (25(OH)D) was determined by an immunoassay on the Abbott Architect analyser (Abbott Laboratories, Abbott Park, Illinois, USA). Assay CVs for PTH were < 5% at PTH concentrations of 41 and 105 ng/L. Assay CVs ranged between 5.0 and 10.7% at serum 25 (OH)D concentrations between 25 and 85 nmol/L. The reference range for PTH is 10–65 ng/L, serum albumin: 40–52 g/L, serum creatinine: 45–84 µmol/L. Serum 25(OH) vitamin D > 50 nmol/L is considered sufficient, 25–50 nmol/L: insufficient and < 25 nmol/L: deficient.

### Statistical analyses

Statistical analysis was performed using IBM SPSS Statistics 26 for Windows (LEADTOOLS©, LEAD Technologies, Inc., USA). Mean and SD were derived for all continuous variables. Between group analyses were carried out using ANCOVA with Bonferroni correction for multiple comparisons. Chi-squared test was used to compare the prevalence of fractures and secondary causes of osteoporosis in the 3 groups. The paired student *t* test was performed to compare baseline and follow-up values in BMD and HSA parameters within each group. Independent *t* test was used to compare change in BMD and HSA parameters between patients with secondary causes of osteoporosis and those without in all 3 groups. The One Sample *t* test was also used to test whether there were any significant difference in % changes in BMD and HSA parameters from baseline (change score between the 2 time points compared to zero) within each group. A “*p*” value < 0.05 was considered statistically significant.

## Results

### Clinical characteristics

Fifty-five female patients, aged 73.0 (8.3) years, had received TPD and were included in Group A. The majority of patients has previously sustained 2 or more fragility fractures (70%). All patients had previously tried or received oral bisphosphonate previous to TPD. At the time TPD was started, 48 patients were on bisphosphonate with a duration of mean [SD] 45.6 [40.8] months, 35 on oral bisphosphonate and 13 were on iv bisphosphonate. One patient had started Dmab for 18 months, 1 on raloxifene for 3 years and 5 (9%) were not on any osteoporosis medications at the time TPD was started. The reasons for switching to TPD were intolerance/contra-indications (*n* = 36) and lack of efficacy (*n* = 15). A significant proportion of patients had secondary risk factors for osteoporosis (*n* = 17). This included current use of glucocorticoids (*n* = 11), rheumatoid arthritis (*n* = 4), connective tissue disease (*n* = 1), inflammatory bowel disease (*n* = 1).

Of the 116 patients (105F, 11 M) aged 72.2(10.8) years) receiving denosumab in Group B, 96 (83%) had sustained 1 or more fragility fractures. Forty-four patients had a secondary risk factor for osteoporosis: 28 patients were either on or had previous exposure to glucocorticoids, rheumatoid arthritis (*n* = 3), systemic lupus erythematosus (SLE) (*n* = 3), endocrine disorders (primary hyperparathyroidism/diabetes mellitus) *n* = 2, treatment with aromatase inhibitors (*n* = 7). One hundred and two (91.8%) patients had previous treatment with bisphosphonates for 78 (55.2) months. Twenty patients (19.6%) had previously received iv zoledronate, the rest (*n* = 82) had been on oral bisphosphonates. Patients were transitioned to denosumab, at least 12 months following their last dose of zoledronate. Fourteen patients (12%) were not on any treatment at the time denosumab was started because of contra-indications including CKD (*n* = 4), patients choice because of perceived lack of efficacy of bisphosphonate (*n* = 5), intolerance (*n* = 4) and difficulty in gaining venous access for 1 patient who had previously been on iv zoledronate. The main reasons for starting denosumab included lack of efficacy (*n* = 41), intolerance and/or contra-indications (*n* = 61), eGFR was less than 35 ml/min in 19 patients (16.4%) and therefore could not continue bisphosphonates. No new fractures occurred whilst on treatment.

Seventy-seven patients (66F, 11 M) aged 68.5 (12.8) years were included in Group C. Sixty five patients (84.4%) had received previous osteoporosis drugs with a mean duration of 38.4 (22.8) months. Fifty-eight patients had received oral bisphosphonates (75.3%). Ten patients (13%) were not on any osteoporosis drugs prior to starting iv ZOL. The clinical indications for iv ZOL were intolerance/contra-indications (*n* = 36) or poor response to oral bisphosphonates defined as continued decrease in BMD and/or a fragility fracture despite treatment with oral bisphosphonates (*n* = 41) following exclusion of new additional clinical risk factors or co-morbidities such as myeloma, malignancy. Secondary clinical risk factors were prevalent in 29 patients. This included use of glucocorticoids (*n* = 16), co-morbidities such as inflammatory disorders; rheumatoid arthritis (*n* = 4), inflammatory bowel disease (*n* = 3), systematic lupus erythematosus (SLE) (*n* = 6), polymyalgia rheumatica (*n* = 1), endocrine disorders; primary hyperparathyroidism (*n* = 2), letrozole (*n* = 1). BMD at the LS and TH were available for 77 patients at year 1 and 2 and 75 patients at year 3. BMD data at the FN were available from the hospital’s picture archiving and communication system (PACS) on 41, 60 and 56 patients after 1, 2 and 3 doses.

### Changes in BMD following treatment with teriparatide, denosumab and iv zoledronate

**Fig. 1:**
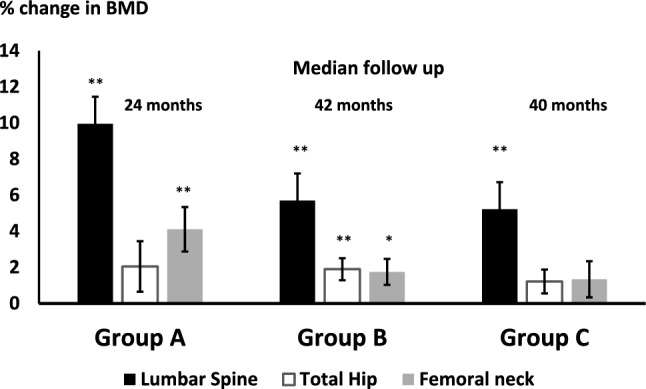
Changes in BMD (mean [SEM]) following treatment with teriparatide (Group A, median follow up 24 months), denosumab (Group B, median follow up 42 months) and iv zoledronate (Group C, median follow up 40 months). ** p < 0.001, * p < *0.05*

A significant increase was seen at the LS, (% change mean [SEM]: 9.9 [1.1] *p* < 0.001) and FN (4.1[1.2] *p* = 0.002) in Group A (TPD). BMD at the LS increased significantly more than at the FN as shown in Fig. 1. In Group B (Dmab), BMD increased significantly at the LS (5.7[0.62] *p* < 0.001), TH (1.9[0.61] *p* = 0.002) and FN (1.75[0.72] *p* = 0.05) (Fig. 1). In group B, BMD at the hip sites were significantly different in patients with CKD stage 4 (*n* = 19) compared to those with e GFR > 30 ml/min (TH % change eGFR < 30 ml/min: -1.86 [1.1], GFR > 30 ml/min: 2.6[0.68] *p* = 0.002, FN % change GFR < 30 ml/min: − 2.2 [1.6], GFR > 30 ml/min: 2.6[0.85] *p* = 0.014). In group C (ZOL), significant increases in BMD at the LS were seen at all time points (% change mean [SEM] 1 year: 3.4[0.62], 2 years: 4.5[0.62] and 3 years: 5.2[0.82], *p* < 0.001). BMD at the TH increased significantly at year 1 and year 2 only (TH (% change mean [SEM] 1 year: 1.1[0.48] *p* = 0.01, 2 years: 2.25[0.46], *p* < 0.001 and 3 years: 1.2[0.68], *p* = 0.08). No significant changes were seen at the FN at any time (% change mean [SEM]1 year: 0.6[0.9], 2 years: 1.6[0.9] and 3 years: 1.3[1.3]), although BMD increased over time. Between group analysis of BMD showed a significant difference in % change in BMD at the LS in Group A compared to Groups B (*p* = 0.003) and C (after 3 doses of ZOL) (*p* = 0.002). No significant differences were seen at the TH and FN. There were no significant differences in changes in BMD at any site between patients with secondary causes of osteoporosis and, those without, in Group A and in C at all time points. In contrast with Group B, the % change in BMD at the TH was significantly lower in patients who had a secondary cause for osteoporosis (TH: % change secondary OP: − 0.12 [0.86], no secondary causes: 3.1[0.81] *p* = 0.007).

### Changes in HSA parameters following treatment with teriparatide, denosumab and iv zoledronate

**Fig. 2:**
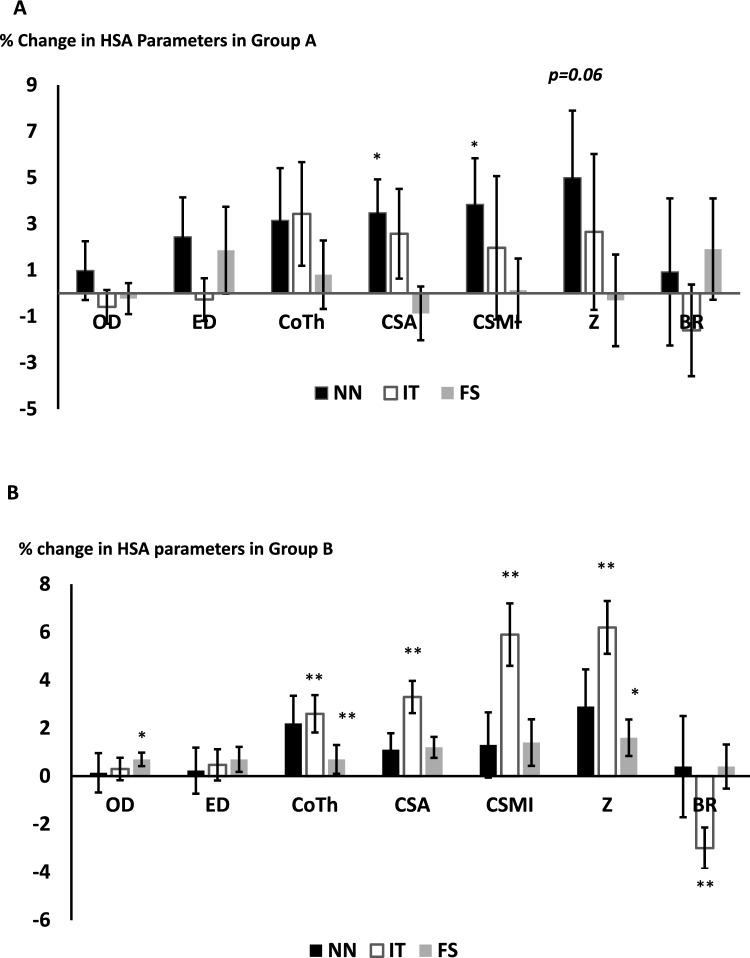
Percentage changes (mean [SEM]) in the HSA parameters at the narrow neck (NN), intertrochanter (IT) and femoral shaft (FS) following treatment with teriparatide (**A**) and denosumab (**B**). ** p < 0.01 * p < *0.05.* HSA parameters (OD subperiosteal (outer) diameter, ED endocortical (inner) diameter, Co Th cortical thickness, CSA cross sectional area, CSMI cross-sectional moment of inertia (CSMI), Z section modulus, BR buckling ratio

In group A (TPD), there were significant increases in CSA at the NN following treatment (% change from baseline (*n* = 55 mean [SEM] CSA: 3.56[1.6] *p* = 0.01) and CSMI: 4.1[1.8] *p* = 0.029). There was a trend in Z at the NN: 5.2[2.7], *p* = 0.06. We did not observe any significant changes in the HSA parameters at the IT or FS. This is shown in Fig. 2A. Section modulus (Z) at the FS was significantly lower in patients with secondary causes of osteoporosis compared to those without (Z: % change secondary OP: − 6.2 [3.4], no secondary causes: 2.1[2.07] *p* = 0.047).

In group B (Dmab) (*n* = 116), significant changes were observed at the IT (% change from baseline mean [SEM] CSA: 3.3[0.67] *p* < 0.001, Co Th: 2.8[0.78] *p* = 0.001, CSMI: 5.9[1.3] *p* < 0.001, Z:6.2[1.1] *p* < 0.001 and BR: − 3.0[0.86] *p* = 0.001).There were no significant changes at the NN. We observed small but significant changes at the FS (OD: 0.7[0.28] *p* = 0.016, CSA: 1.2[0.44] *p* = 0.005, Z: 1.6[0.76] *p* = 0.04) as shown in Fig. 2B. The % change in OD, ED, CSA were lower in patients with CKD stage 4 (e GFR < 30 ml/min) at the NN, although the results failed to reach significance (Table 2). At the IT, significant reduction in Co Th was seen in patients with CKD (% change CKD Co Th: − 1.9 [2.1], e GFR > 30 ml/min: 3.5[0.81], *p* = 0.025). There were differences between the 2 groups in CSA and BR, although the results failed to reach significance (% change CSA CKD: − 0.2[2.1], e GFR > 30 ml/min: 3.9[0.67] *p* = 0.07), BR CKD: 1.9[2.11], e GFR > 30 ml/min: − 3.9[0.82], *p* = 0.07). At the FS, there were significant changes between the 2 groups in Co Th, CSA, Z and BR (% change Co Th CKD: − 2.8 [1.6], GFR > 30 ml/min: 1.4[0.62] *p* = 0.026, CSA; CKD: − 0.83[1.1], GFR > 30 ml/min: 1.64 [0.46] *p* = 0.05, Z CKD: -1.2[1.3], GFR > 30 ml/min: 2.1[0.87], *p* = 0.036, BR CKD: 4.7[2.3], GFR > 30 ml/min: − 0.42 [0.98], *p* = 0.05) with a trend at the ED (% change ED; CKD: 3.1[1.46], GFR > 30 ml/min: 0.28[0.54], *p* = 0.08). There were significant differences in some HSA parameters at the FS in patients with secondary causes of osteoporosis compared to those without (ED: % change secondary OP: 2.1 [0.87], no secondary causes: − 0.1[0.63] *p* = 0.043, CSA: % change secondary OP: 0.04[0.72], no secondary causes: 2.0[0.53] *p* = 0.035, Co Th: % change secondary OP: − 1.2[1.0], no secondary causes: 1.84[0.73] *p* = 0.016, BR: % change secondary OP: 2.9[1.64], no secondary causes: − 1.1[1.06] *p* = 0.041).

**Table 2:** % change in HSA parameters with Dmab in CKD v/s non-CKD patients, *p ≤ *0.05*, #*p* = *0.07*

% Change mean (SEM)	eGFR < 30 ml/min *n* = 19	e GFR > .30 ml/min *n* = 97	*p* value
Narrow neck (NN)			
Outer diameter (OD)	− 0.98 (2.29)	0.36 (0.89)	*0.59*
Endocortical diameter (ED)	− 0.79 (2.56)	0.43 (1.03)	*0.67*
Cross-sectional area (CSA)	− 0.65 (1.56)	1.40 (0.76)	*0.25*
Cortical thickness (CoTh)	2.03 (3.67)	2.24 (1.18)	*0.95*
Cross sectional moment of inertia (CSMI)	5.9 (3.12)	0.45 (1.51)	*0.13*
Section modulus (Z)	11.1 (6.72)	1.32 (1.27)	*0.17*
Buckling ratio (BR)	1.90 (2.99)	− 3.90 (0.83)	*0.68*
Intertrochanter (IT)			
Outer diameter (OD)	2.31(1.64)	− 0.033(0.46)	*0.18*
Endocortical diameter (ED)	2.72(1.8)	0.03(0.68)	*0.17*
Cross-sectional area (CSA)	− 0.2(2.07)	3.94(0.68) ^#^	*0.07*
Cortical thickness (CoTh)	− 1.89(2.11)	3.51(0.81) *	*0.025*
Cross sectional moment of inertia (CSMI)	6.09(4.7)	5.86(1.3)	*0.96*
Section modulus (Z)	3.26(3.55)	6.82(1.17)	*0.35*
Buckling ratio (BR)	1.90(2.99)	− 3.90(0.83) ^#^	*0.07*
Femoral Shaft (FS)			
Outer diameter (OD)	1.47(0.76)	0.57(0.3)	*0.29*
Endocortical diameter (ED)	3.1(1.46)	0.28(0.54)	*0.085*
Cross-sectional area (CSA)	− 0.84(1.11)	1.64(0.46) *	*0.026*
Cortical thickness (CoTh)	− 2.8(1.64)	1.38(0.62) *	*0.05*
Cross sectional moment of inertia (CSMI)	− 0.07(1.66)	1.72(1.12)	*0.38*
Section modulus (Z)	− 1.25(1.3)	2.12(0.87) *	*0.036*
Buckling ratio (BR)	4.72(2.34)	− 0.42(0.98)*	*0.05*

**Fig. 3:**
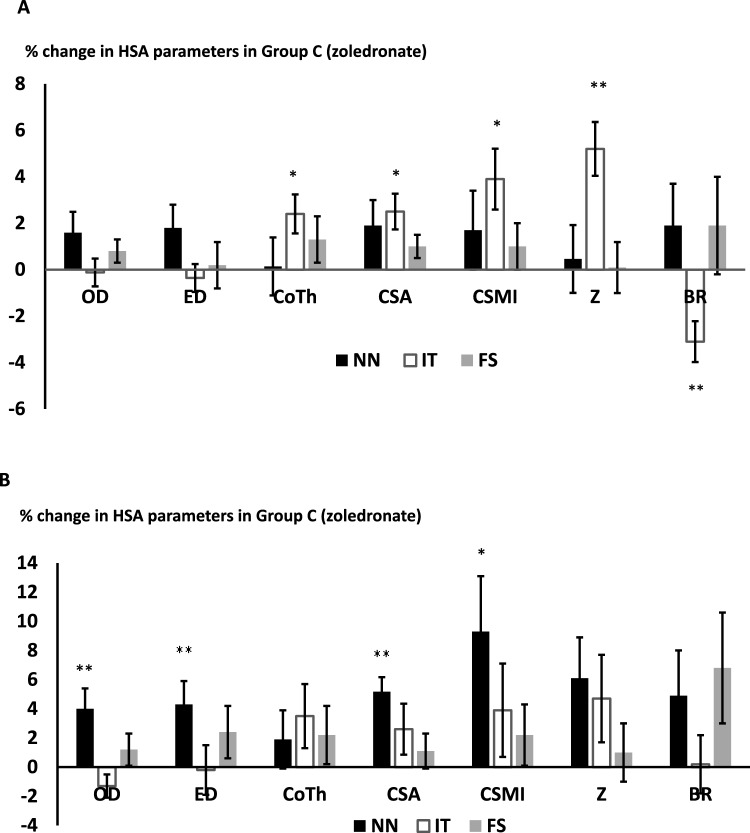
Percentage changes (mean [SEM]) in the HSA parameters at the narrow neck (NN), intertrochanter (IT) and femoral shaft (FS) following treatment with zoledronate after 2 doses (**A**) and after 3 doses (**B**). ** p < 0.01 * p < *0.05.* HSA parameters (OD subperiosteal (outer) diameter, ED endocortical (inner) diameter, Co Th cortical thickness, CSA cross sectional area, CSMI cross-sectional moment of inertia (CSMI), Z section modulus, BR buckling ratio

In group C (ZOL) (*n* = 75), there were no significant changes in HSA parameters following 1 dose of ZOL. Following 2 doses of iv zoledronate, significant changes were seen at the IT only (% change from baseline mean [SEM] CSA: 2.5[0.77] *p* = 0.017, Co Th: 2.4[0.84] *p* = 0.012, CSMI: 3.9[1.3] *p* = 0.017, Z:5.2[1.16] *p* < 0.001 and BR: − 3.1[0.88] *p* = 0.001) as shown in Fig. 3A. Following 3 doses of ZOL, significant changes were observed at the NN (% change from baseline mean [SEM] OD: 4.0[1.4] *p* = 0.0005, ED: 4.3[1.67] *p* = 0.009, CSA: 5.2[1.8] *p* = 0.003, CSMI:9.3[3.8] *p* = 0.019 as shown in Fig. 3B. There were no significant differences in changes in the HSA parameters in patients with secondary causes of osteoporosis compared to those without in Group C following the first, second and third dose of ZOL at the NN and IT. At the FS, after 3 doses of ZOL, changes in OD and ED were significantly larger in those with secondary causes of osteoporosis (OD: % change secondary OP: 1.94[1.08], no secondary causes: -0.83[0.84] *p* = 0.047, ED: % change secondary OP: 4.17[2.14], no secondary causes: − 1.32[1.26] *p* = 0.032). Between-group analysis in the whole population showed no significant differences in the % change in the HSA parameters at the NN, IT or FS between Group A, Group B and Group C (following 3 doses of ZOL).

## Discussion

In summary, there was significant improvement in BMD, particularly at the lumbar spine with all 3 osteoporosis medications. Changes in the HSA parameters varied between the 3 groups. Significant improvement in hip geometric and mechanical parameters was seen with denosumab and zoledronate, at the NN and IT regions. Our data suggest that derivation of the HSA parameters from DXA acquired images provide additional information in the evaluation of the mechanical implications of changes in BMD in routine clinical care setting.

Larger changes in BMD observed following TPD (Group A) were found at the LS compared to the FN and TH, due to differences in trabecular and cortical bone composition at these latter sites [18, 19]. The greater improvement in BMD at the LS following TPD compared to Dmab or ZOL relates to TPD’s anabolic effects on trabecular bone. Improvement in BMD was also observed following treatment with Dmab (Group B) as previously described in treatment naïve patients [4]. The changes in BMD at the hip sites were more pronounced in patients without any secondary risk factors and whose e GFR was > 35 ml/min as a result of the opposing effects of mineral disturbances in CKD stage 4/5 which affect bone metabolism and mineralisation. Changes in BMD following ZOL could be seen as early as after the first dose, particularly at the LS spine as previously documented [3]. Changes in BMD at the hip sites in our study were more modest and may be related first to previous uses of oral bisphosphonate as patients did not have a washout period before switching to zoledronate, second a significant proportion of patients had secondary risk factors and thirdly, BMD data at the FN was available in a sub-group of patients only.

HSA parameters derived from DXA scan acquired images may provide mechanically important geometric effects that underlie changes in BMD [13, 14]. Several meta-analyses have shown that teriparatide reduces the risk of hip fracture by 56% [6]. The importance of hip geometry and structure is crucial in the understanding the effect of TPD on hip strength. The cross-sectional area of mineralised cortical and trabecular bone (CSA) and CSMI was increased at the NN following TPD and would indicate increased resistance to axial loading and bending forces. There was a trend in improvement in section modulus (Z) at the NN implying improved resistance to bending. Although other studies have shown increases in OD and Co Th at the femoral neck [10], these changes were not significant in our study and may be related to the small study number and the presence of other risk factors or co-morbidities as would be expected in a ‘real-life’ setting. We did not observe any significant detrimental changes in the geometric and mechanical parameters at the FS, a cortical rich site which can be subject to the ‘cortical steal effect of intermittent PTH [20, 21]. However, the presence of secondary risk factors did alter the response to TPD, particularly on section modulus (Z) at the FS. Bone quality is impaired in patients with secondary osteoporosis [22, 23]. Our study thus extends these findings to reduced treatment responsitivity to TPD, particularly in cortical bone compartment in these patients. This merits further investigations.

In contrast to TPD, treatment with Dmab led to significant changes in HSA parameters at the IT and FS, resulting in improvement in Co Th and CSA and in mechanical parameters. These results demonstrate the impact of Dmab on bone strength at the IT where focal thinning of the bone has been associated with trochanteric fracture [24]. These effects have been previously reported, although improvement was at all 3 sites [16]. Other studies have reported that increases in cortical mass density and Co Th following 3 years treatment with Dmab was more pronounced in the trochanteric region which would be in agreement with our study [25]. Dmab has been shown to reduce the risk of wrist fractures and this has been attributed to its positive effect on cortical thickness and strength at the radius which is a cortical rich site like the FS [26]. This would explain, in part, our findings of a positive effect of Dmab at the FS. There may be several explanations why we did not observe any effects at the NN including that our patients were not treatment naïve and had previously been treated with bisphosphonates. The cohort also included a small number of patients with CKD stage 4 and with secondary risk factors which can influence bone quality. Although Dmab has been shown to improve bone quality in secondary osteoporosis, this was mainly at the LS [27]. Our results show an attenuation of Dmab effects on the HSA parameters in secondary osteoporosis, particularly at the FS although this may be driven by the effects of CKD on cortical bone [28].

Although ZOL and Dmab are potent anti-resorptive agents, their mode of action differ [7, 29]. Oral bisphosphonates have been shown to improve HSA parameters with the largest effects seen at the IT region and lesser effects at the FS [15]. Alendronate’s effects on hip geometry are smaller than Dmab, particularly at the IT and FS [9]. Similar changes to Dmab were seen at the IT after 2 doses of ZOL, suggesting an improvement in bending strength, axial strength and cortical stability at the IT. These findings have been previously described in the ZONE study although the effect of ZOL on hip geometry and biomechanical parameters was seen at all 3 sites [30]. The lack of improvement at the NN, in particular, in our study may be explained by previous treatment with oral bisphosphonates. Following 3 doses of zoledronate significant increases in OD, ED, CSA and CSMI at the NN were seen. Increases in OD were more prominent in the patients with secondary risk factors and may be secondary to the bone loss seen at the endosteal surface and increased ED as a compensatory mechanism to maintain bone bending strength and resistance to loading in this sub-group [31]. Additional factors may relate, in part, to the increasing age and frailty although there was no control group to compare.

This study has several limitations. Firstly, this was a retrospective study and we do not have control groups for each osteoporosis medications. BMD and HSA measurements were not done at fixed time points compared to the stringent rules of a randomised controlled trial. However, the results regarding certain HSA parameters are similar to what has been previously reported given that the patients were not treatment-naïve and had secondary risk factors in some cases. In addition, the HSA methodology has some inherent limitations as it is based on certain assumptions on bone shape and tissue mineralisation. However, good correlations have been found between HSA derived bone geometry at the hip and QCT [32, 33]. Femoral positioning between scans had already been assessed as being correct by experienced DXA technologists when the DXA scan images were acquired for BMD measurements.

HSA parameters derived from DXA can provide unique information on the therapeutic efficacy of commonly prescribed osteoporosis medications and underlie their effects on bone strength and their anti-fracture mechanisms. Among the parameters contributing to bone quality, OD, Co Th and biomechanical parameters at the hip such as section modulus (Z) and buckling ratio (BR) have been shown to be independent predictors of hip fracture [13, 14]. These parameters may be introduced in clinical practice to assess bone strength and treatment response. This technique can thus have a supplemental role in clinical situations where it is important to evaluate bone strength considering not only BMD but also bone quality. However, further prospective clinical studies using newer scanners with better improved software, image resolution and algorithms are needed before the introduction of DXA HSA in routine clinical practice.

In conclusion, our study shows that using DXA scan-derived HSA parameters of hip geometry, treatment with TPD, Dmab and ZOL had important effects on some biomechanical parameters associated with bending and axial strength in different areas of the hip. This methodology may be used in the clinical setting to provide further information on the anti-fracture efficacy of osteoporosis drugs.

## Data Availability

The non-identifiable/anonymised data that support the findings of this study are available from the corresponding author, [GH], upon reasonable request by clinical or academic researchers for non-commercial use.
